# Vaccinators' Perception of HPV Vaccination in the Saa Health District of Cameroon

**DOI:** 10.3389/fpubh.2021.748910

**Published:** 2022-01-10

**Authors:** Eposi Haddison, Afizu Tambasho, Gael Kouamen, Randolph Ngwafor

**Affiliations:** ^1^District Health Service, Saa, Cameroon; ^2^Central Technical Group-Expanded Programme on Immunisation, Ministry of Public Health, Yaounde, Cameroon; ^3^Regional Delegation of Public Health, Bafoussam, Cameroon; ^4^Biotechnology Centre, University of Yaounde 1, Yaounde, Cameroon

**Keywords:** HPV–human papillomavirus, vaccination, Cameroon, perception, vaccinators

## Abstract

**Introduction:** Cervical cancer is the second most prevalent cancer among women in Cameroon. In November 2020, the HPV vaccine was introduced into the expanded programme on immunisation. However, uptake of the vaccine has been slow in the Centre region as opposed to other regions in the country. We therefore sought to describe vaccinators' perception of HPV vaccination in Saa health district.

**Methods:** A self-administered questionnaire with both open-ended and closed questions was used to assess the perception of HPV vaccination among 24 vaccinators from the Saa health district. Quantitative data were summarised as proportions while qualitative data were deductively and inductively coded and thematically analysed.

**Results:** Most vaccinators (75%, *n* = 18) had a good knowledge about cervical cancer and HPV vaccination. Fourteen (58.3%, *n* = 14) vaccinators correctly reported the target group, number and spacing of doses for the HPV vaccine. Fourteen (58.3%) vaccinators favoured HPV vaccination while the others (*n* = 10) were sceptical. Sceptics felt manufacturers hadn't given enough proof of the safety of the vaccine and lacked confidence in government's assessment of the epidemiological situation. The COVID 19 pandemic, fear of infertility and the negative influence of social media were perceived as the main reasons for community hesitancy. Vaccinators criticised health authorities for failing to sensitise the population about the HPV vaccine before its introduction.

**Conclusion:** Vaccinators' perceptions of HPV vaccination may influence the offer of HPV vaccination services. Measures to increase acceptance of HPV vaccination and ownership of the activity among vaccinators have to be put in place.

## Introduction

According to the World Health Organisation (WHO), Cervical cancer is the fourth most common cancer among women globally despite the disease being preventable and curable (1). Consequently, cervical cancer has been earmarked for elimination by 2030 (1). To achieve the elimination target by 2030, WHO has spelt out three goals for countries to achieve; a) 90% of girls should be fully vaccinated by the age of 15 years; b) 70% of women screened using a high-performance test by the age of 35 years, and again by the age of 45 years and; c) 90% of women with pre-cancer treated and 90% of women with invasive cancer managed. Cervical cancer is caused by human papilloma viruses (HPV) which are transmitted through sexual contact, with the most oncogenic types being HPV 16 and 18 (2).

Compared to high-income countries, the burden of HPV infection is more prevalent in low-income countries (LICs) (2). In LICs, access to screening and treatment is limited thus highlighting the importance to vaccinate young girls if elimination is to be achieved (3). The WHO recommends vaccination of girls aged 9–14 years old because they are considered not yet sexually active and therefore, prior to HPV exposure which makes this period the best for vaccination against the cancer-causing virus (2). The HPV vaccine became available in 2006 (4) and globally as of May 2020, 52 countries had carried out demonstration projects and 128 had introduced the vaccine into their National Immunisation Programmes (NIP) (5). Forty-one additional countries were scheduled to introduce the HPV vaccine into their NIP by 2023.

In Cameroon, cervical cancer is the second most common cancer among women (6) with about 2,356 estimated new cases diagnosed annually (6). Doh et al., in 2017 reported an HPV prevalence of 36% in the country (7) with HPV types 16, 18 and 45 being reported to be the most prominent in several settings (8, 9). In the Centre region of Cameroon, cervical cancer is the most frequently reported cancer (10). Low socio-economic status, HIV infection and multiple partners, have been shown to be risk factors for HPV infection in Cameroon (8, 11). The 2020–2024 National Strategic Plan for Prevention and Cancer Control has a specific objective two that aims to increase primary prevention interventions by 25%, through communication for behaviour change in favour of cancer control. One of the priority interventions to achieve this objective is immunisation with the HPV vaccine. Several demonstration projects carried out in the country between 2010 and 2016 targeting 9- to 13-year-old girls revealed a high acceptability of the vaccine (12) and the NIP's capacity to accommodate the vaccine. Thus, with Gavi, the Vaccine Alliance's support, the HPV vaccine was scheduled to be introduced into the NIP in November 2020. The objective was to fully vaccinate 70% of 9-year-old girls by 2021 using the school and fixed strategies. Pre-introductory activities included microplanning, capacity building of stakeholders, vaccine procurement and community sensitisation.

Specifically, in the Saa health district of the Centre region, microplanning for HPV introduction took place in September 2020 with primary school teachers, chief of health areas and community health workers (CHWs) in attendance. The microplanning from the workshop brought about the enumeration of 1,003 girls aged 9 years fully registered in 61 primary schools in the district. Vaccination was scheduled to take place during the week of the 23^rd^ of November 2021. That same month, a capacity building workshop for providers was organised. Out of 27 vaccinators invited, only 15 participated in the training. Therefore, health area chiefs were tasked with capacitating all vaccinators in their zone who didn't attend the training.

Quadrivalent Gardasil was the vaccine to be administered in two doses with a 6-month interval between doses. Each vial contained a single dose of the vaccine. Based on the estimated number of 9-year-old girls in the district, 940 doses of the vaccine were allocated to the district to be supplied as need be. The first batch of vaccines were transported from the regional cold chain to the district cold chain 1 month prior to vaccine introduction. Due to the lack of electricity in the health district, all vaccines were stored in the district cold chain and only supplied to vaccination centres on vaccination days. Communication material were also distributed to the various vaccination centres.

During the week of the 23^rd^ of November 2020 only 10 girls were vaccinated. After carrying out an inquiry among vaccinators and CHWs in the district, the reasons given for non-vaccination were: teachers didn't inform parents about the vaccination schedule, parents refused their children should be vaccinated, vaccinators didn't follow up with schools to ensure consent forms were sent to parents nor did they schedule any vaccination date, the Catholic church had warned its congregants not to allow their children get vaccinated among others. Faced with these, vaccinators were instructed to reschedule vaccination dates in each school and ensure teachers sent written consent forms to parents. CHWs were instructed to continue community sensitisation. Despite these measures, only 43 girls were vaccinated between January to March 2021 using both the school and fixed strategy. A similar trend was observed at regional level whereby only 294 out of 50,840 girls had been vaccinated between January and March 2021 thus making the Centre region the second least performing region in Cameroon. On the other hand, the Southwest region which is characterised by socio-political crisis and displaced populations was ranked the best performing region with 3,024 out of 18,525 girls receiving their first dose of the vaccine during the same period.

As a result of this low uptake, an advocacy meeting was held between health district authorities and all primary school teachers in Saa health district. The teachers were sensitised on cervical cancer and the importance of the vaccine. They were also urged by their hierarchy to inform parents about the vaccine. Similarly, another capacity building workshop on HPV vaccination was carried out for all vaccinators in the district. Despite the workshop, no orders were placed for the vaccine by vaccinators at the district cold chain. Faced with this situation, we therefore sought to evaluate vaccinators' perception of HPV vaccination in order to design and improve strategies to increase uptake of the HPV vaccine in the district. We also aimed to provide some insight as to why coverage may be low in the region.

## Methods

Saa health district has 30 health facilities out of which 26 offer vaccination services. Vaccination sessions are usually organised by the head nurse who may be assisted by one or two support staff. All 30 vaccinators in the district were invited to take part in the study. A pre-tested self-administered paper-based questionnaire which contained both open-ended and closed questions was used to assess each participant's perception of HPV vaccination. The questionnaire was designed specifically for the study then sent to five vaccinators out of the district and two subject matter experts who checked for comprehension, coherence and acceptability of the questions. Adjustments were made based on their feedback before administration to study participants.

The questionnaire (Supplementary Material 1) covered two main components; knowledge on HPV and HPV vaccine and perception toward HPV vaccination. To assess the knowledge of HPV and HPV vaccines, questions asked were centred on the definition, transmission, prevention and treatment of HPV as well as the target group for HPV vaccination as per the Expanded Programme on Immunisation (EPI) in Cameroon. Knowledge was graded on 11 points (a point for each question). For this study, a score of nine and above was considered as good knowledge, 6–8 as average knowledge and five and below as poor knowledge. Questions on perception focused on HPV vaccine service delivery, perceived reasons for community hesitancy, perception on the necessity and safety of HPV vaccine and self-perceived ability to persuade parents to accept HPV vaccination.

Quantitative data were entered into Excel then exported in Stata 14 for analysis. Quantitative data were summarised as proportions. Using a deductive and inductive approach, responses to open ended questions were coded and the codes then categorised under three main themes that were: a) vaccinators' perception of HPV vaccination; b) vaccinators perceived reasons for community hesitancy and; c) vaccinators perceived commitment toward HPV vaccination. Furthermore, several sub-themes emerged relating to vaccinators' perceptions of the HPV vaccination programme in the Saa district. Coding was carried out by HE then verified by KG.

## Results

A total of 30 vaccinators were invited to take part in the study out of which 24 participated. The participants comprised of 15 females and nine males, among which 17 were state registered nurses, six nurse aids and one biomedical engineer (Table 1). The ages of the participants ranged from 28 to 58 years with a median age of 46.5 years. Years of service as a vaccinator ranged from 1 to 30 years with a median duration of 9.5 years. A majority (80%) of the vaccinators were Catholic.

**Table 1:** Socio demographic characteristics of vaccinators.

**ID**	**Age category**	Sex^*^	**Grade**^≠^****	**Number of years as a vaccinator**	**Religion**
V1	45–54	M	SRN	≥16	Catholic
V2	25–34	F	SRN	6–10	Protestant
V3	45–54	F	SRN	11–15	Protestant
V4	25–34	F	NA	6–10	Christian
V5	45–54	F	SRN	11–15	Christian
V6	35–44	F	SRN	6–10	Catholic
V7	45–54	F	SRN	11–15	Catholic
V8	55–64	M	SRN	≥16	Catholic
V9	45–54	F	NA	11–15	Catholic
V10	55–64	F	NA	≥16	/
V11	25–34	M	SRN	1–5	Catholic
V12	35–44	F	BME	1–5	Catholic
V13	35–44	F	NA	11–15	Catholic
V14	25–34	M	SRN	1–5	Catholic
V15	45–54	F	SRN	≥16	Catholic
V16	45–54	F	NA	11–15	Catholic
V17	25–34	M	SRN	6–10	Catholic
V18	45–54	M	SRN	≥16	Catholic
V19	55–64	M	NA	≥16	Catholic
V20	25–34	M	SRN	1–5	Catholic
V21	45–54	F	SRN	≥16	/
V22	55–64	M	SRN	≥16	Catholic
V23	25–34	F	SRN	1–5	/
V24	35–44	F	SRN	1–5	Christian

* *M, male; F, female*.

≠ *SRN, State registered nurse; NA, Nurse aid; BME, Biomedical engineer*.

### Knowledge of HPV and HPV Vaccination

All the vaccinators had heard about HPV and the HPV vaccine (Table 2). We found that 75% (n=18) of the vaccinators had a good knowledge of HPV and HPV vaccination, 12.5% (n=3) had an average knowledge and the remaining 12.5% had poor knowledge. Among the vaccinators 58.3% (n=14) gave the right number of HPV vaccine doses to be administered, the interval between the doses and the reason why the target group of 9-year-old girls was chosen.

**Table 2:** Vaccinators' responses to questions on HPV knowledge and perception (*n* = 24).

**Question**	**Correct response**	**Wrong response**
**A Knowledge on HPV and HPV vaccination**		
Have you heard about HPV?	24	0
What does H.P.V. stand for?	19	5
How is HPV transmitted	18	6
What disease can an individual infected with HPV have	19	5
Can an individual infected with HPV be treated?	14	10
Have you heard about the HPV vaccine?	24	0
What does the HPV vaccine protect against?	22	2
In Cameroon who is the target for the vaccine?	19	5
Why do you think this group is considered a target for the HPV vaccine?	15	9
How many doses have to be administered and at what interval?	16	8
What vaccine strategies have been adopted for HPV vaccination?	20	4
**Question**	**Yes**	**No**
**B. Perception about HPV vaccination**		
Have you been in contact with schools in your catchment area to offer the HPV vaccine?	16	8
Do you think the HPV vaccine is necessary for Cameroonians?	18	6
In your opinion is the HPV vaccine safe?	14	10
In your opinion, do you think there are other vaccines that are safer than the HPV vaccine?	9	14
In your opinion, do you think there are other vaccines that are less safe than the HPV vaccine?	9	14
Do you go out of your way to convince parents to vaccinate their children with HPV?	18	6
Do you feel you are being pressured by hierarchy to administer the HPV vaccine?	14	10
Do you feel you are properly equipped to convince parents to accept the HPV vaccine?	7	17

### Perceived Reasons for Community Hesitancy

A majority of the vaccinators (62.5%, *n* = 15) had been in contact with schools in their catchment area to provide the HPV vaccine. However, only two vaccinators declared having vaccinated up to 50% of their target population. When asked about the reasons for hesitancy, three main reasons emerged.

#### Coronavirus Disease 2019

Vaccinators felt that information surrounding the emergence, spread and management of COVID 19 was the main reason parents and guardians refused to have their children vaccinated. Some vaccinators felt the timing for introduction of the vaccine was not good. The community had not been adequately sensitised before introduction of the vaccine hence the confusion with COVID 19.

*Did they have to wait for COVID 19 to introduce the vaccine? That's why people are refusing (Vaccinator 9)*

Others stated that the community believed the vaccine was being used as a cover by pharmaceutical industries to infect them with the coronavirus and also as a means to make money. According to them the government was capable of maliciously infecting them with the coronavirus. There was the fear of potentially losing their children if they were vaccinated.

*Parents feel this way because of the unexplained arrival of COVID 19 and they say whites want to multiply the cases in Africa as it is in their country (Vaccinator 2)*.

#### Infertility

Many vaccinators reported that the community had strong socio-cultural beliefs and saw the vaccine as a threat to procreation. The community believed the vaccine will render their children sterile hence tarnishing their reputation or bringing an end to their lineage.

*They say that the vaccine will sterilise their girls and their generation will not be continued due to this vaccine (Vaccinator 14)*.

*If in reality it protects girls against cervical cancer, it's preferable that we accept it. Africans don't like sterile women (Vaccinator 1)*.

#### Influence of Social Media

According to vaccinators social media played a big role in parents' attitude toward HPV vaccination. Vaccinators felt parents and guardians believed all the fake news and rumours about the HPV vaccine going around on social media and were not willing to accept the right information.

*Parents and guardians are in contact with school authorities who have been intoxicated with wrong information on HPV. (Vaccinator 12)*

### Perception of HPV Vaccination

We found two types of vaccinator attitudes toward HPV vaccination.

#### Favoured Vaccination

Fourteen (58.3%) vaccinators favoured HPV vaccination. They all felt the vaccine was safe and necessary for Cameroonians. Their assurance was based on the fact that the vaccine had undergone several tests and had been deemed safe by the international scientific committee. Moreover, the government couldn't authorise a vaccine that wasn't safe for its population. They reported that the prevalence of cervical cancer in the country warranted the introduction of the HPV vaccine in order to protect the community.

*The vaccine is considered safe because it has been subjected to a lot of testing for its quality. (Vaccinator 6)*

*Several women suffer from cervical cancer and lose their lives so it's necessary to vaccinate to avoid these deaths. (Vaccinator 11)*

#### Sceptics

Ten (41.7 %) vaccinators either reported that the vaccine was unsafe or they were not certain of its safety and/or it was not necessary for Cameroonians. These vaccinators were classified as sceptics. Some vaccinators reported that there was not enough evidence from manufacturers to show that the vaccine was safe whereas others said they were not properly empowered to judge if a vaccine was safe and only time will tell. With regards to the necessity of the vaccine, some sceptics considered that the prevalence of cervical cancer in Cameroon was too low to warrant introduction of the HPV vaccine into the immunisation schedule.

*No experience with vaccine quality. A vaccine that has raised a lot of controversy. (Vaccinator 17)*

### Perceived Commitment for Vaccination

Each vaccinator's commitment for vaccination was based on perceived roles and responsibilities. The more they felt a task belonged to another party, the more critical they were of that party.

#### Personal Commitment

Most of the vaccinators (75%, *n* = 18) reported that they did their best to convince parents to vaccinate their girls against HPV. However, only seven vaccinators felt they were sufficiently equipped to carry out the task. A few recognised that they were responsible for sensitising the population on the HPV vaccine and proposed strategies to do so.

#### Public Health Authorities

A majority (75%, *n* = 18) of the vaccinators felt public health authorities had not played their role in properly sensitising the population on the HPV vaccine before its introduction. Fourteen vaccinators (58.3%) felt they were being pressured by hierarchy to administer the HPV vaccine. According to them hierarchy always asked for monthly reports for HPV vaccination. Therefore, this placed a burden on them to sensitise parents and guardians sometimes at the cost of their safety.

*Let the regional and district management teams go into the field to sensitise the population, let them explain the advantages and disadvantages of the vaccine. Let the district management team themselves go into the field to ensure implementation and create awareness among the population. (Vaccinator 16)*

## Discussion

This study revealed that 75% of vaccinators had a good knowledge of cervical cancer and the HPV vaccine. However, 41.7% of vaccinators were sceptical about the HPV vaccine. Vaccinators considered community hesitancy was as a result of the fear of COVID 19, infertility and adherence of the population to fake rumours about the vaccine on social media. A majority of the vaccinators criticised the government for not adequately preparing them and the population to receive this new vaccine.

This study identified a good level of knowledge among vaccinators. Not only were all vaccinators aware of the vaccine, a majority had the adequate knowledge required to offer the HPV vaccine. The level of knowledge among vaccinators in this study was higher than those reported in other settings. Ndizeye et al., in 2015 reported low knowledge of HPV vaccination knowledge among physicians in Burundi (13). Similarly, Edu et al., in 2021 reported suboptimal levels of knowledge among nurses and midwives in a hospital in Ghana (14). Limited access to training on HPV and cervical cancer prevention have been cited as a main reason for poor knowledge among health workers in African settings (13, 14). Access to such trainings improve knowledge as seen in our setting. Similarly, a study carried out by Wamai et al., during the demonstration phase in Cameroon in 2013 showed that nurses at the demonstration sites had a good knowledge about HPV vaccination (15). Capacity building of health workers empowers them with the necessary tools to provide services confidently and engage the community to use these services.

Vaccinators reported that some parents hesitated to have their girls vaccinated due to the risks of contracting COVID 19. Since the outbreak of COVID 19, a drop in the health seeking behaviour of communities has been reported in several settings (16–18). People fear contracting the SARS-COV-2 at health facilities. In Cameroon, the government's response to the COVID 19 pandemic has worsened the population's trust of in the health sector (19–21). Though containment of the virus has allowed the resumption of mass campaigns, the fear of contracting the SARS-COV-2 through health personnel still persists. Also, the COVID 19 vaccine is being perceived by the population as a potential source of infection. Though not yet introduced in the country at the time of the HPV vaccine introduction, the administration of the new HPV vaccine amid rumours of the introduction of a COVID 19 vaccine led to confusion among the population with some parents preferring to abstain from all vaccines.

Vaccinators also perceived the fear of infertility as a major barrier to HPV vaccination. In Africa, infertility is highly stigmatised (22). In some cases, it may affect marital life, lead to promiscuity, exposure to sexually transmitted infections and psychological trauma especially for women (22). This fear of infertility has been reported to affect reproductive health (RH) decision making practises among populations (23). They abstain from vital reproductive health services such as family planning, screening and vaccination. In our setting this fear of infertility was also expressed by vaccinators and can be considered a major factor to HPV vaccine hesitancy. More efforts in proximity as well as mass sensitisation on the role RH services play in improving fertility are needed to improve uptake of RH services such as HPV vaccination. This sensitisation will also help dissipate rumours which are being spread on social media. The affordability of android phones and the availability of the internet even in remote villages has facilitated access to social media. Unfortunately, most people are not able to philtre true from fake news and are prone to believe rumours which tend to be more sensational than the truth.

Despite the fact that the Vicar general of the diocese covering the district had instructed all Catholic schools, health facilities and congregants not to accept the vaccine based on his personal convictions, none of the vaccinators reported this as one of the reasons for hesitancy. However, the health district service considered this as a major cause of hesitancy as access into Catholic schools for vaccination was denied nor were HPV vaccination schedules announced in Catholic churches. Religious leaders have been reported to influence the health behaviour of their congregants both on a personal and community level (24). This key role is used as a tool for health promotion in communities (25). Despite this influence, the final decision rests with parents and some parents use religion as a reason not to have their children vaccinated (26). In 2017 the Pontifical Academy for Life stated that all clinically recommended vaccines could be used with a clear conscience (27) therefore, continuous advocacy with local Catholic church leaders on the safety and necessity of the HPV vaccine is needed since they greatly influence the health behaviour of the population.

Health providers' trust in the health system is likely to shape their recommendation practises. A considerable number of vaccinators in our setting mistrusted health authorities and pharmaceutical companies thus rendering them sceptical about the HPV vaccine. Vaccinators are less likely to recommend HPV vaccination if they do not see its necessity nor alleviate parents' worries about the vaccine (28). These directly affect HPV vaccination services provision and uptake. Reasons given for the general state of mistrust of health workers in Cameroon toward the state include poor working conditions, low salaries, pressure from hierarchy to perform, poor communication among others (20, 29). In Cameroon communication during health campaigns is carried out at all levels of the health pyramid. The central level engages mass media while the district level handle proximity sensitisation (30, 31). For the introduction of the HPV vaccine, vaccinators felt the central level didn't adequately play its part in sensitising the population via mass media thereby rendering proximity sensitisation very tough. However, it has been shown that in rural areas during vaccination campaigns, a majority of the population is sensitised through proximity sensitisation as opposed to mass media (32, 33). Generally, the district performs well in routine and supplementary immunisation activities. The district registered a BCG vaccination coverage of 81.2%, a DTP3 coverage of 83.6% and a specific dropout rate of 7.9 % in 2020. Similarly, a measles rubella campaign in 2019 pre introduction of the second dose of the measles rubella vaccine into the EPI registered a coverage of 98% among children aged 12 to 59 months. Also, the district registered a coverage of 96.6% in 2021 during the polio SIA targeting children 0–59 months which was carried out during the COVID pandemic. In contrast the HPV vaccine had only registered a coverage of 5.4% seven months post introduction. Vaccinators need to acknowledge the essential role they play in sensitising parents about the HPV vaccine and creating an enabling environment for vaccine uptake. Nonetheless, the state still needs to play its role in communicating adequately about the HPV vaccine especially in the COVID 19 context where a lot of financial and human resources have been diverted from other health programs to fight against the pandemic.

This study had several limitations. Firstly, the study targeted only vaccinators so a complete view of the reasons for community hesitancy could not be obtained as no young girls or parents were questioned. Secondly, the study used open ended questions as opposed to interviews thus vaccinators may not have expressed all their feelings. Lastly, due to the small target population size, some meaningful data may have been lost with those who declined to participate, however, we are confident that the data obtained from vaccinators gives an insight of their perception toward the HPV vaccine.

## Conclusion

Uptake of HPV vaccination in the Saa health district has been very low 8 months post its introduction. Despite a good knowledge of cervical cancer and the HPV vaccine among vaccinators, a majority remain sceptical about the vaccine. This scepticism may influence the recommendation rate of the vaccine thus leaving young girls exposed to HPV and cervical cancer. Measures to improve acceptance and ownership of the HPV vaccination programme among vaccinators are needed.

## Data Availability Statement

The original contributions presented in the study are included in the article/Supplementary Material, further inquiries can be directed to the corresponding author.

## Ethics Statement

Since this study was carried out as part of routine EPI activities in the district, ethical approval for this study and written informed consent from the participants of the study were not required in accordance with local legislation and national guidelines. Nonetheless, the objective of the study was explained to vaccinators and emphasis was laid on the anonymity of responses. Verbal consent was obtained from each vaccinator who agreed to take part in the study.

## Author Contributions

EH conceived the study and collected data. EH and GK analysed the data. All authors wrote, read, and approved the final manuscript.

## Conflict of Interest

The authors declare that the research was conducted in the absence of any commercial or financial relationships that could be construed as a potential conflict of interest.

## Publisher's Note

All claims expressed in this article are solely those of the authors and do not necessarily represent those of their affiliated organizations, or those of the publisher, the editors and the reviewers. Any product that may be evaluated in this article, or claim that may be made by its manufacturer, is not guaranteed or endorsed by the publisher.
