# Supplementary material for: Vaccinators' Perception of HPV Vaccination in the Saa Health District of Cameroon
Source: Front Public Health. 2022 Jan 10;9:748910. doi: 10.3389/fpubh.2021.748910 (PMC8784768; doi:10.3389/fpubh.2021.748910)
Supplement: Supplementary file 1 [file Table_1.DOCX]

REPUBLIQUE DU CAMEROUN Paix – Travail – Patrie ----------------------

MINISTERE DE LA SANTE PUBLIQUE ------------------------ DELEGATION REGIONALE DU CENTRE

**-------------------------**

SERVICE DE SANTE DU DISTRICT DE SA’A

REPUBIC OF CAMEROON

Peace – Work - Fatherland

---------------------

MINISTRY OF PUBLIC HEALTH

------------------------ CENTER REGIONAL DELEGATION

**-------------------------**

SA’A DISTRICT HEALTH SERVICE

**HPV VACCINE HESITANCY IN SAA HEALTH DISTRICT**

*Please fill in the blank spaces and put a tick where necessary.*

1. **SOCIO-DEMOGRAPHIC CHARACTERISTICS**
2. Age: ………… 2. Sex: ………. 3. Years of service: …………

4. Years as a vaccinator: ………. 5. Religion: ………. 6. Qualification……………

1. **HPV KNOWLEDGE**
2. Have you heard about HPV before? Yes……. No……
3. What does H.P.V. stand for? …………………………………….
4. How is HPV transmitted? ………………………………………………………………………………………………
5. What disease can an individual infected with HPV have? ……………………………….
6. Can an individual infected with HPV be treated? Yes…… No……
7. Have you heard about the HPV vaccine? Yes……. No……
8. What does the HPV vaccine protect against? …………………………….…………..
9. In Cameroon who is the target for the HPV vaccine? ……………………………………
10. Why do you think this group is considered a target for the HPV vaccine?

..............................................................................................................................................

1. How many HPV vaccine doses have to be administered and at what interval? ………………………………………………………………………………………………
2. What vaccine strategies have been adopted for HPV vaccination in Cameroon?

School……….. Door to door……… Fixed……… Outreach………

1. **PERCEPTION OF THE HPV VACCINE ADMINSTRATION**
2. How often do you organise routine vaccination sessions?

Everyday……… Once a week………. Twice a week……. Once a month……… Twice a month……

1. During these sessions do you administer the HPV vaccine? Yes…… No……..
2. Do you know your target population for the HPV vaccine? Yes…… No…….
3. What is your target? < 50……. 50-100…… 100-200……… >200…..
4. Have you been in contact with schools in your catchment area to offer the HPV vaccine?

Yes……….. No……….

1. If no, why not? ……………………………………………………………………………
2. If yes, were you able to vaccinate up to 50% of your target population? Yes….. No…..
3. What are some of the reasons given for refusal?

………………………………………………………………………………………………………………………………………………………………………………………………………………………………………………………………………………………………………………………………………………………………………………………………

1. Why do you think parents/guardians feel that way? .............................................................................................................................................

…………………………………………………………………………………………….

…………………………………………………………………………………………….

1. In your opinion are some of the reasons given in question 8 and 9 valid?

Yes…….. No……….

1. If yes, which ones?

………………………………………………………………………………………………………………………………………………………………………………………………………………………………………………………………………………………………

1. Why do you think the reasons are valid or not valid?

………………………………………………………………………………………………………………………………………………………………………………………………

………………………………………………………………………………………………

1. Do you think the HPV vaccine is necessary for Cameroonians?

Yes……. No……. Not really…….

Please justify your answer

…………………………………………………..………………………………….………………………………………………………………………………………………………

………………………………………………………………………………………………

1. In your opinion is the HPV vaccine safe? Yes…… No………

Please justify your answer

…………………………………………………………………………………..

………………………………………………………………………………………………………………………………………………………………………………………………

1. In your opinion, do you think there are other vaccines that are safer than the HPV vaccine?

Yes……. No……..

1. If Yes, which vaccines do you think are safer?.....................................................................
2. In your opinion, do you think there are other vaccines that are less safe than the HPV vaccine?

Yes…….. No………

1. If Yes, which vaccines do you think are less safe?...............................................................
2. What considerations did you make to regard these vaccines as safe or less safe than the HPV vaccine?

................................................................................................................................................................................................................................................................................................

1. Do you go out of your way to convince parents to vaccinate their children with HPV?

Yes…….. No……….

Please justify your answer

……………………………………………………………………..…………………………………………………………………………………………………………………….

1. Do you feel you are being pressured by hierarchy to administer the HPV vaccine?

Yes……. No……….

Please justify your answer

……………………………………………………………………………………………………………………………………………………………………………………………………………………………………………………………………………………………....

1. Do you feel you are properly equipped to convince parents to accept the HPV vaccine?

Yes……. No………

1. In your opinion what can be done to improve HPV vaccine acceptability in Saa?.

………………………………………………………………………………………………………………………………………………………………………………………………………………………………………………………………………………………………………………………………………………………………………………………………………………………………………………………………………………………………

**THANKS FOR YOUR PARTICIPATION**
